# Racial disparities in total ankle arthroplasty utilization and outcomes

**DOI:** 10.1186/s13075-015-0589-2

**Published:** 2015-03-21

**Authors:** Jasvinder A Singh, Rekha Ramachandran

**Affiliations:** Medicine Service, Birmingham VA Medical Center, Faculty Office Tower 805B, 510 20th Street S, Birmingham, AL 35294 USA; Department of Medicine at the School of Medicine and Division of Epidemiology at the School of Public Health, University of Alabama, Faculty Office Tower 805B, 510 20th Street S, Birmingham, AL 35294 USA; Department of Orthopedic Surgery, Mayo Clinic College of Medicine, 200 1st St SW, Rochester, MN 55905 USA

## Abstract

**Introduction:**

The objective of this study was to examine the racial disparities in total ankle arthroplasty (TAA) utilization and outcomes.

**Methods:**

We used the National Inpatient Sample (NIS) to study the time-trends. Race was categorized as White and Black. Utilization rates were calculated for the U.S. general population per 100,000. Hospital length of stay, discharge disposition and mortality after TAA were assessed. We used the Cochran Armitage trend test to assess time-trends from 1998 to 2011 and chi-square test to compare TAA utilization. We used analysis of variance or chi-squared test to compare the characteristics of Whites and Blacks undergoing TAA and logistic regression to compare mortality, length of stay and discharge to home versus medical facility.

**Results:**

The mean ages for Whites undergoing TAA were 62 years and for Blacks was 52 years. Significant racial disparities were noted in TAA utilization rates (/100,000) in 1998, 0.14 in Whites vs. 0.07 in Blacks (*P* < 0.0001; 2-fold) and in 2011, 1.17 in Whites vs. 0.33 in Blacks (*P* < 0.0001; 4-fold). Racial disparities in TAA utilization increased significantly from 1998 to 2011 (*P* < 0.0001). There was a trend towards statistical significance for the difference in the length of hospital stay in Blacks vs. Whites (52.9% vs. 44.3% with length of hospital stay higher than the median; *P* = 0.08). Differences in the proportion discharged to an inpatient medical facility after TAA, 16.6% Blacks vs. 13.4% Whites, were not significant (*P* = 0.36).

**Conclusions:**

This study demonstrated significant racial disparities with lower TAA utilization and suboptimal outcomes in Blacks compared to Whites. Further studies are needed to understand the mediators of these disparities and to assess whether these mediators can be targeted to reduce racial disparities in TAA.

**Electronic supplementary material:**

The online version of this article (doi:10.1186/s13075-015-0589-2) contains supplementary material, which is available to authorized users.

## Introduction

Racial and ethnic disparities are widespread in health care [1-5]. The 2002 Institute of Medicine report called for ‘Confronting Racial and Ethnic Disparities in Health Care’ [6]. The 2009 Institute of Medicine’s comparative effectiveness research agenda included reduction in racial disparities in the first quartile of the 100 top priority areas [7]. Unless all segments of the population receive optimal health care, best health care outcomes cannot be realized for the population as a whole. Racial disparities in arthroplasty are of great interest, due to several reasons. Lower extremity osteoarthritis (OA), the most common reason for arthroplasty, has similar or higher prevalence in Blacks compared with Whites [8]. OA is more severe and more symptomatic in Blacks than Whites [9-13]. Arthroplasty, a definitive surgical treatment for end-stage OA (and other types of arthritis), is associated with significant improvements in quality of life and functional ability [14,15]; and there is no evidence that benefits of arthroplasty are lower in Blacks, compared with Whites. This implies that the need for arthroplasty is at least as great or greater in Blacks, when compared with Whites. Additionally, the benefits of arthroplasty do not seem to differ by race to justify a lower utilization in Blacks compared with Whites. However, numerous studies have shown that the utilization of knee or hip arthroplasty is much lower in Blacks than Whites [16-19]. This indicates that Blacks with end-stage arthritis are not experiencing the arthroplasty-associated quality-of-life improvements to the same extent as Whites. More studies are therefore needed to understand the extent and reasons for racial disparities in arthroplasty utilization.

Arthroplasty involves replacing diseased joint surfaces with plastic or metal components to relieve pain and improve joint function and mobility. Hip and knee joints are the most common lower extremity arthroplasty surgeries performed for end-stage arthritis. Another lower extremity joint affected not uncommonly is the ankle joint. Ankle joint arthritis can lead to disabling pain and functional limitation [20]. Total ankle arthroplasty (TAA) is the surgical treatment for end-stage ankle joint disease [21,22], an alternative to ankle arthrodesis [23]. The increasing use of TAA is due to much better clinical and radiographic outcomes with the second-generation and third-generation prostheses compared with the earlier designs [24,25].

In a recent study, we found an increase in TAA utilization in the United States from 1998 to 2011 [26]. As the utilization of TAA increases, an important question is whether the racial disparities described for knee and hip arthroplasty exist for TAA. To our knowledge, no studies have assessed racial disparities in TAA utilization and outcomes. Our study objectives were to assess racial disparities in TAA utilization and outcomes. We asked whether there are any racial disparities in utilization of TAA, whether utilization disparities between Blacks and Whites are decreasing over time, and whether the outcomes of TAA differ by race.

## Methods

### Data source

We used the National Inpatient Sample (NIS; previously called Nationwide Inpatient Sample) data from 1998 to 2011 for this study. The NIS is the largest inpatient care database that provides data on hospital inpatient stays for all patients in the United States, and is publicly available. The NIS contains data from approximately 8 million hospital stays each year. The US NIS contains approximately a 20% stratified sample of US community hospitals; in 2011, 46 states provided data [27]. The NIS includes all patients regardless of the payer. In other words, the NIS includes patients covered by Medicare, Medicaid, private insurance and the uninsured. Since the NIS has a representative sample, it has been used to analyze national trends in outcomes of various conditions during the inpatient stay [28,29]. The NIS can be weighted to obtain national estimates. The NIS contains primary and secondary diagnoses, patient demographics, length of stay, admission and discharge status, hospital characteristics and total charges. For each hospital admission, the International Classification of Diseases Ninth Revision Common Modification (ICD-9-CM) diagnostic and procedure information are available. The Institutional Review Board at the University of Alabama at Birmingham approved the study. No patient consent was needed, since these data are de-identified.

### Study cohort, time periods, main predictor and covariates

The study cohort consisted of patients who underwent a primary TAA, identified by the presence of ICD-9-CM procedure code 81.56 during the hospitalization, from 1998 to 2011. We divided the calendar years into 2-year to 3-year intervals *a priori* (1998 to 2000, 2001 to 2002, 2003 to 2004, 2005 to 2006, 2007 to 2008 and 2009 to 2011), in order to have an adequate number of cases for analyses for mortality, discharge status and length of hospital stay (since the groups with smaller numbers, Black males and Black females, had few or no patients with outcomes in some years).

Race, the main independent variable of interest, was categorized as White and Black. We included covariates previously shown or suspected to be associated with arthroplasty utilization and/or outcomes [30-35]. Patient demographic (age, sex) and clinical characteristics (underlying diagnosis, comorbidity) were obtained. We categorized age into <50 years, 50 to <65 years, 65 to <80 years and ≥80 years, and the underlying diagnosis into OA, rheumatoid arthritis, avascular necrosis of the bone, fracture and other (post-traumatic arthritis, septic arthritis, Charcot joint, and so forth). Comorbidity was assessed using the validated Deyo-Charlson index [26], a sum of 17 comorbidities [27,28] at the index admission preoperatively, based on the presence of ICD-9-CM codes. The Deyo-Charlson index was categorized as 0, 1 or 2 or more.

### Study outcomes

The TAA utilization rates for Whites and Blacks were calculated by dividing the respective NIS estimates by the total US population for Whites/Blacks for the respective year obtained from the US census site, expressed per 100,000 patients [36]. Hospital length of stay was calculated as the number of days from admission to discharge for the index TAA admission. Mortality related to the index admission was assessed using the mortality during the index hospital admission. Discharge status was categorized as discharge: home with or without home health care; or to an inpatient facility that included a short-term hospital, a skilled nursing facility, an intermediate care facility or another type of inpatient facility. We excluded patients who died in the hospital or left the hospital against medical advice from the analyses of utilization and discharge outcomes only. Outcomes (hospital stay, discharge disposition and mortality) were calculated for categories of 2 or 3 years for time-trend analyses. We categorized the annual hospital volumes for TAA as <5 procedures/year, 5 to 9 procedures/year, 10 to 14 procedures/year, 15 to 24 procedures/year and ≥25 procedures/year. Length of stay was categorized into greater than the median or less than the median.

### Statistical analyses

We calculated 95% confidence intervals for utilization rates based on binomial proportions. We applied data weights as recommended to obtain weighted estimates [37]. We used the Cochran Armitage trend test to assess time trends from 1998 to 2011 and the chi-square test to compare TAA utilization between 1988 and 2011. Analysis of variance or the chi-squared test was used to compare the characteristics of Whites and Blacks undergoing TAA. Logistic regression was used to compare mortality, length of stay and discharge to home versus medical facility.

## Results

### Patient characteristics

In total, 12,122 Whites and 488 Blacks underwent TAA during the study period. The mean ages for the Whites and Blacks were 61.9 and 52.0 years, respectively; 55% and 62% were female, respectively (Table 1). The underlying diagnosis was OA in 62.5% of Whites and 47.2% of Blacks. A total 5% of Whites and 11.2% of Blacks had a Deyo-Charlson index ≥2 (Table 1). A significantly higher proportion of Blacks (47.6%) than Whites (35.2%; *P* = 0.04) underwent TAA at a hospital with an annual volume <5 TAA procedures (Table 1).

**Table 1:** **Characteristics of patients undergoing total ankle arthroplasty, 1998 to 2011**

	**White**	**Black**	***P*** **value**
	**(** ***n*** **= 12,122)**	**(** ***n*** **= 488)**	
Age (years)	61.9 (61.1, 62.7)	52.0 (49.6, 54.3)	<0.0001
Female	54.8	61.9	0.19
Age group			
< 50 years	15.5	42.2	<0.0001
50 to 64 years	38.3	43.6	0.28
65 to 79 years	41.2	12.5	<0.0001
≥ 80 years	5.1	1.7	0.11
Underlying diagnosis			
Osteoarthritis	62.5	47.2	0.005
Rheumatoid arthritis	5.6	10.3	0.04
Fracture	0.1	1.0	0.04
Traumatic arthropathy	18.8	18.6	0.96
Avascular necrosis	0.5	0.9	0.51
Other	12.6	22.0	0.01
Deyo-Charlson score			
0	71.8	65.8	0.25
1	23.2	22.9	0.96
≥ 2	5.0	11.2	0.01
Hospital volume (procedures/year)			
< 5	35.2	47.6	0.04
5 to 9	25.6	26.5	0.89
10 to 14	12.3	5.7	0.14
15 to 24	17.7	14.4	0.42
≥ 25	9.3	5.8	0.21

Data presented as mean (95% confidence interval) or percentage.

### Time trends in TAA utilization by race

**Figure 1:**
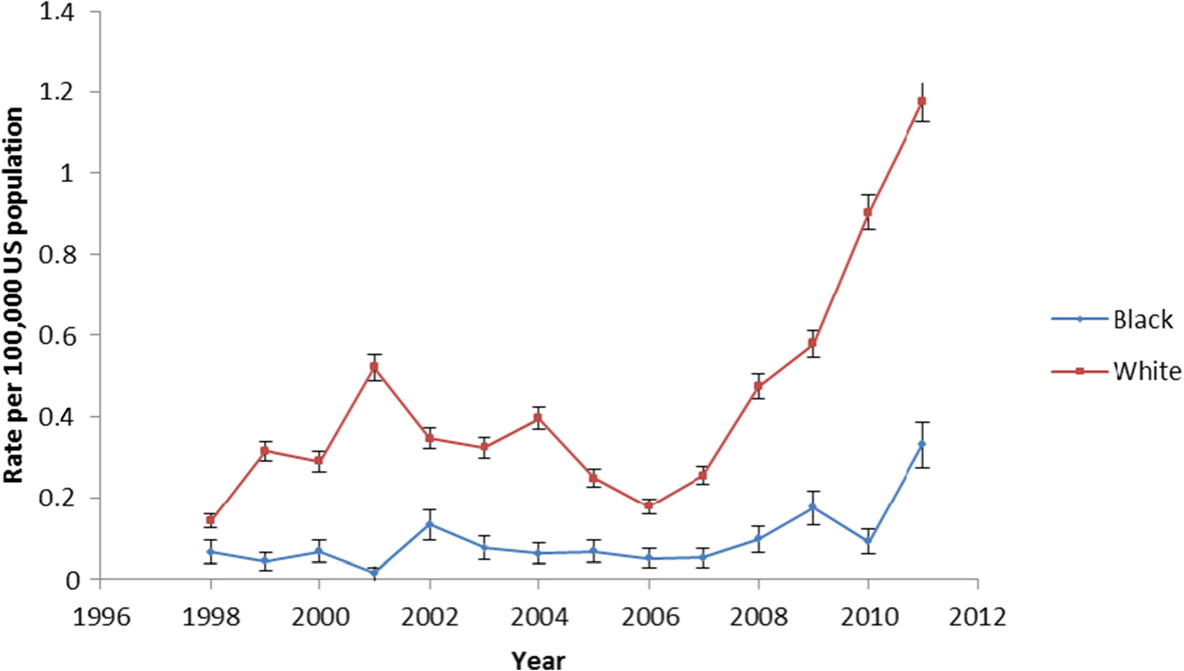
**Total ankle arthroplasty utilization rates per 100,000 in Whites and Blacks over time.**

We compared the time trends in TAA utilization by race. TAA utilization was significantly higher in Whites than Blacks in 1998, 0.14 versus 0.07 per 100,000 (*P* <0.0001; twofold rate) (Table 2 and Figure 1). In 2011, TAA utilization was 1.17 in Whites and 0.33 in Blacks, showing significant racial disparities (*P* <0.0001; 3.5-fold rate). Disparities in TAA utilization by race increased statistically significantly from 1998 to 2011 (*P* <0.0001). The twofold higher rate in Whites compared with Blacks in 1998 increased to an almost fourfold higher rate in 2011 (Table 2 and Figure 1). The absolute White–Black difference in TAA utilization rate was 0.07 per 100,000 in 1998 and 0.84 per 100,000 in 2011 (a 12-fold higher difference).

**Table 2:** **Time trends in annual total ankle arthroplasty utilization by race**

**Year**	**White**	**Black**	***P*** **value**
1998	0.14	0.07	<0.0001
1999	0.32	0.04	<0.0001
2000	0.29	0.07	<0.0001
2001	0.52	0.01	<0.0001
2002	0.35	0.13	<0.0001
2003	0.33	0.08	<0.0001
2004	0.40	0.06	<0.0001
2005	0.25	0.07	<0.0001
2006	0.18	0.05	<0.0001
2007	0.26	0.05	<0.0001
2008	0.47	0.10	<0.0001
2009	0.58	0.18	<0.0001
2010	0.90	0.09	<0.0001
2011	1.17	0.33	<0.0001

Total ankle arthroplasty utilization rates increased from 1998 to 2011: White, *P* <0.0001; Black, *P* <0.0001 (Cochran Armitage trend test for time trends). White–Black disparity was significantly more in 2011 compared with 1998: *P* <0.0001.

In further analyses of Whites and Blacks by sex, we found that the utilization rates differed significantly between the groups in 1998 and 2011, numerically slightly higher in females than males (Table 3). In addition, the rates increased significantly from 1998 to 2011 in White males (*P* <0.0001), White females (*P* <0.0001), Black males (*P* = 0.0031) and Black females (*P* <0.0001).

**Table 3:** **Time trends in total ankle arthroplasty utilization rates per 100,000 by race and gender**

	**White males**	**Black males**	**White females**	**Black females**	***P*** **value**
1998	0.11	0.07	0.17	0.06	<0.0001
1999	0.36	0.09	0.27	0	N/A
2000	0.29	0	0.29	0.13	N/A
2001	0.49	0	0.56	0.03	N/A
2002	0.28	0.06	0.41	0.20	<0.0001
2003	0.25	0.11	0.39	0.05	<0.0001
2004	0.34	0.03	0.45	0.10	<0.0001
2005	0.22	0.03	0.28	0.10	<0.0001
2006	0.16	0.11	0.19	0	N/A
2007	0.22	0.04	0.29	0.06	<0.0001
2008	0.42	0.05	0.53	0.14	<0.0001
2009	0.57	0.19	0.59	0.16	<0.0001
2010	0.84	0.05	0.97	0.12	<0.0001
2011	1.13	0.25	1.22	0.41	<0.0001

N/A, not applicable since a frequency of 0 for at least one category did not allow calculation of a *P* value; a frequency of 0 indicates that no TAA procedure was performed in that group in that calendar year. Cochran Armitage trend test for time trend from 1998 to 2011: White males, *P* <0.0001; Black males, *P* = 0.0031; White females, *P* <0.0001; and Black females, *P* <0.0001. The p-value in the table indicates the difference between the groups.

**Figure 2:**
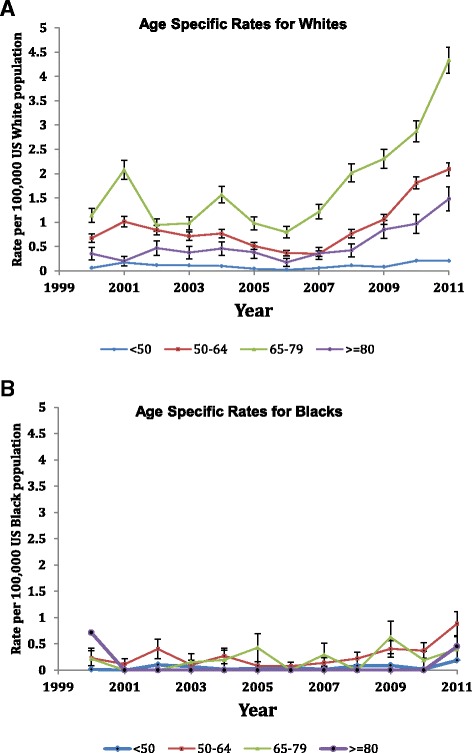
**Age-specific total ankle arthroplasty utilization rates per 100,000 for Whites and Blacks. (A)** Age-specific rates for Whites. Cochran Armitage test comparing total ankle arthroplasty (TAA) utilization in all years: age <50, *P* = 0.11; age 50 to 64, *P* <0.0001; age 65 to 79, *P* <0.0001; age ≥80, *P* <0.0001. **(B)** Age-specific rates for Blacks. Cochran Armitage test comparing TAA utilization in all years: age <50, *P* = 0.07; age 50 to 64, *P* = 0.053; age 65 to 79, *P* = 0.009; age ≥80, *P* = 0.0002.

A significant increase in TAA utilization over time (1998 to 2011) was seen for all four age groups in Whites except for <50 years (*P* = 0.11 for <50 years and *P* < 0.0001 for all others; Figure 2). When comparing 1998 with 2011, TAA utilization increased significantly for all age groups (*P* <0.0001). In Blacks, no significant time-related increase in TAA utilization rates was seen in the <50 or 50 to 64 age categories (*P* = 0.07 and 0.053) and slight increases in the 65 to 79 and ≥80 age groups (*P* = 0.009 and 0.0002; Figure 2). Additional file 1 shows these time trends in utilization by race in more detail. Over time, we noted an increase in age and comorbidity, and change in the underlying diagnosis.

### Outcomes variation by race

The proportion of Black patients with hospital stay longer than the median stay was higher than White patients (52.9% vs. 44.3%), with a trend towards significance (*P* = 0.08; Table 4). A slightly higher proportion of Blacks compared with Whites were discharged to an inpatient facility after TAA (16.6% vs. 13.4%), but this was not significant (*P* = 0.36). Five Whites and four Blacks died after TAA; mortality was higher in Blacks compared with Whites (*P* = 0.02; Table 4). Additional file 2 shows these outcomes by time period in more detail. In general, the length of stay decreased and discharge to an inpatient facility increased over time.

**Table 4:** **Unadjusted outcomes by race including all data from 1998 to 2011**

	**White (** ***n*** **= 12,122)**	**Black (** ***n*** **= 488)**	***P*** **value**
Length of stay > median	5,366 (44.3%)	258 (52.9%)	0.08
Discharge			
Home	10,476 (86.6%)	403 (83.4%)	0.36
Inpatient facility	1,628 (13.4%)	80 (16.6%)	
Mortality	5 (0.04%)	4 (0.9%)	0.02

Data presented as *n* (%).

## Discussion

To our knowledge, our study is the first to examine racial disparities in TAA utilization and outcomes in the United States. We found important differences in demographic and clinical characteristics of Blacks and Whites undergoing TAA. The TAA utilization rates were lower in Blacks compared with Whites for all years. White–Black disparities in TAA utilization increased over the 12-year period, from a twofold higher rate in Whites compared with Blacks in 1998 to an almost fourfold higher rate in 2011, significant both statistically and clinically (absolute difference in rate increased 12-fold). Blacks had a longer hospital stay and higher mortality compared with Whites, but no differences in discharge to home versus an inpatient facility. Several findings from our study merit further discussion.

The TAA utilization rate was significantly lower in Blacks compared with Whites in 1998 as well as 2011. The absolute White–Black difference in TAA utilization rate was 0.07 per 100,000 in 1998 and 0.84 per 100,000 in 2011 (a 12-fold higher difference) and the relative rate was twofold in 1998 and fourfold in 2011. Racial disparities increased significantly, both statistically and in a clinical meaningful way. Our time-trend analysis is the first to indicate that there was clear evidence of widening racial disparity in TAA utilization over the 12-year study period. Racial disparities with lower rates of knee and hip arthroplasty utilization in Blacks, compared with Whites, have been shown in studies using regional data [38] and national Medicare data [18]. Our study extended these findings of the presence of racial disparities to TAA populations. The increasing disparities in TAA utilization should be very concerning, since this trend was far worse than an absence of reduction of disparities in the utilization over time for knee/hip arthroplasty, reported previously [39,40]. Policy-makers should consider the worsening racial disparities in TAA utilization rates an important warning. This trend of worsening disparities is in the opposite direction to that recommended by the 2012 Institute of Medicine report [6], which drew attention to existing racial disparities in health care and the need to reduce and eliminate disparities. We found an increasing gap in TAA utilization between Blacks and Whites over time. Our study findings of increasing disparity indicate that policy-makers need to develop and implement policies to eliminate these disparities, including the widening gap in TAA utilization between Blacks and Whites.

The lower arthroplasty utilization rates in Blacks could not be explained by surgical appropriateness or disease burden, since there are no significant racial differences in proportions with at least moderately severe OA deemed appropriate for arthroplasty [41]. Blacks have a higher disease burden of arthritis than Whites [8-13]. Socioeconomic status differences explained only some racial differences in arthroplasty utilization [42]. Blacks have a higher fear of arthroplasty compared with Whites [43]. Previous studies showed that Blacks presented with worse preoperative hip and knee function at the time of hip or knee arthroplasty than Whites [44] and had longer delays to presentation and higher body mass index at the time of hip arthroplasty compared with Whites [45]. This indicated a worse preoperative patient status in Blacks undergoing arthroplasty, which is associated with poorer postoperative pain and function outcomes [46]. Blacks were less willing than Whites to undergo total knee or hip joint replacement surgery [47]. Future studies are needed to explore whether patient preferences, health care access barriers, adequacy of social support/network and/or other factors can explain these racial disparities. A better understanding of racial disparities and factors associated with it will help the development of interventions targeting these racial disparities in arthroplasty utilization. With our findings in TAA, these findings may be particularly relevant to Blacks undergoing TAA. Once effective interventions can be developed, they can be implemented to eliminate these racial disparities.

We found that Blacks undergoing TAA differed from Whites regarding demographic and clinical characteristics. Blacks undergoing TAA were younger than Whites, had a higher proportion of females, had differences in the underlying diagnosis (lower proportion had OA, higher proportion had rheumatoid arthritis and other diagnoses) and had higher comorbidity. Since the impact of each of these characteristics (age, sex, diagnosis and medical comorbidity) on TAA outcomes is not known at this time, it is not possible to predict whether these factors positively or negatively impact the outcomes of TAA in Blacks versus Whites. Future studies that can examine the impact of these factors on outcomes can help us better understand their contribution to differences in TAA outcomes or the lack thereof.

We found that a higher proportion of Blacks who underwent TAA had hospital stay longer than the overall median hospital stay compared with Whites, which almost reached significance (*P* = 0.056). Mortality after TAA, although rare in both Blacks and Whites, was higher in Blacks compared with Whites. These are novel findings. In the absence of any previous studies, no comparisons could be made. This longer hospital stay in Blacks compared with Whites might be due to a delayed presentation [45], worse preoperative pain and function [44], and worse function on performance tests [13] in Blacks, all of which can delay post-arthroplasty recovery.

Our study showed that Blacks were also more likely to undergo TAA at hospitals with the lowest annual volume (<5 procedures/year) compared with Whites. This racial difference in utilization of hospitals with lower volume may have contributed to worse outcomes among Blacks compared with Whites after TAA, including a longer post-arthroplasty hospital stay and higher mortality in Blacks. Racial differences in social support, higher comorbidity and higher rates of other risk factors for poor outcomes and mortality might also have contributed. This finding is similar to a recent finding by Cai and colleagues that Blacks undergoing knee/hip arthroplasty were more likely than Whites to be admitted to hospitals with higher risk-adjusted postoperative rates of complications or mortality [48]. Studies need to explore why minorities are more likely to undergo arthroplasty at low-volume hospitals, which are often associated with worse outcomes. The practical implications of these findings are that Blacks need education resources to become knowledgeable regarding the association of hospital volume and arthroplasty outcomes to make informed decisions.

Our study findings must be interpreted considering the study limitations and strengths. The NIS treats each hospital admission as an event, which would theoretically miss bilateral TAAs performed during a single hospitalization. Bilateral TAA is rare, however, so this is unlikely to have biased the estimates. The NIS does not include federal facilities such as military and Veterans Affairs hospitals. However, it is a nationally representative dataset and several studies using NIS data for knee, shoulder and hip arthroplasty have been published [49-52], supporting its generalizability. We used the entire US population for calculating the rates, a method that has been used in other studies from the NIS [53,54]. We used the ICD-9-CM for identifying TAA, similar to the codes used in other arthroplasty studies, which have been found to be accurate [55]. Another limitation of these analyses is that the underlying diagnoses of OA/rheumatoid arthritis/avascular necrosis and so forth may or may not be joint specific; their accuracy and positive predictive values have not been established and therefore some misclassification is possible. Mortality differences were based on very small numbers and therefore should be interpreted with caution.

## Conclusions

This study found that the racial disparity in annual TAA utilization increased from twofold in 1998 to fourfold in 2011 in the United States. Blacks also had a slightly longer hospital stay and higher mortality compared with Whites. There were no racial differences in discharge disposition. The persisting and perhaps worsening racial disparities in TAA utilization and outcomes over a 13-year study period should serve as a wake-up call for patients, surgeons and policy-makers. Further studies are needed to investigate why the racial disparities in TAA are worsening and to develop strategies for addressing this problem. Policy-makers should implement policies that can reduce this racial disparity gap in utilization and outcomes of TAA.

## Additional files

Additional file 1:
**Is a table presenting the characteristics of patients undergoing TAA by race across years in the study period.** Comparisons of TAA utilization in Whites and Blacks over the study period from 1998 to 2010 and change in Whites and Blacks from the first (1998 to 2000) to the last (2009 to 2010) study period. Additional file 2:
**Is a table presenting TAA outcomes by race from 1998 to 2010.** Comparisons of outcomes (mortality, discharge disposition and length of hospital stay) after TAA in Whites and Blacks over the study period from 1998 to 2010 and the change in Whites and Blacks from the first (1998 to 2000) to the last (2009 to 2010) study period.

## Abbreviations

ICD-9-CM: International Classification of Diseases Ninth Revision Common Modification
NIS: National Inpatient Sample
OA: osteoarthritis
TAA: total ankle arthroplasty
