# Supplementary material for: Racial disparities in total ankle arthroplasty utilization and outcomes
Source: Arthritis Res Ther. 2015 Mar 21;17(1):70. doi: 10.1186/s13075-015-0589-2 (PMC4392624; doi:10.1186/s13075-015-0589-2)
Supplement: Additional file 1: — Is a table presenting the characteristics of patients undergoing TAA by race across years in the study period. Comparisons of TAA utilization in Whites and Blacks over the study period from 1998 to 2010 and change in Whites and Blacks from the first (1998 to 2000) to the last (2009 to 2010) study period. [file 13075_2015_589_MOESM1_ESM.docx]

Additional files:

Additional file 1. Characteristics of patients undergoing TAA by race across years in the study period from 1998 to 2010

|  | 1998-2000 | | 2001-2002 | | 2003-2004 | | 2005-2006 | | 2007 - 2008 | | 2009-2010 | | %Change  Last period- first period | | p-value comparing first and last period | |
| --- | --- | --- | --- | --- | --- | --- | --- | --- | --- | --- | --- | --- | --- | --- | --- | --- |
|  | W | B | W | B | W | B | W | B | W | B | W | B | W | B | W | B |
| Mean age (min, max) | 59 (0, 83) | 55 (24, 86) | 60 (19, 88) | 46 (15, 62) | 61 (11, 85) | 49 (32, 67) | 63 (23, 89) | 50 (29, 70) | 63 (28, 90) | 51 (38, 76) | 63 (19, 88) | 55 (36, 73) | 7.0 | 0 | 0.0001 | 0.98 |
| Female (%) | 50 | 58.3 | 57.0 | 81.8 | 60.1 | 54.5 | 56.1 | 44.4 | 57.5 | 69.2 | 53.5 | 55.0 | 7.0 | -5.7 | 0.33 | 0.85 |
| Age group (%) |  |  |  |  |  |  |  |  |  |  |  |  |  |  |  |  |
| <50 | 23.4 | 33.3 | 22.2 | 54.5 | 18.8 | 45.4 | 9.2 | 55.6 | 14.3 | 46.1 | 11.7 | 30 | -50.1 | -10 | <0.0001 | - |
| 50-64 | 34.8 | 50 | 37.4 | 45.4 | 38.6 | 36.4 | 39.9 | 22.2 | 32.0 | 38.5 | 42.1 | 50 | 20.9 | 0.00 | 0.0381 | - |
| 65-79 | 37.9 | 8.3 | 37.1 | 0 | 37.6 | 18.2 | 45.1 | 22.2 | 49.0 | 15.4 | 40.4 | 20 | 6.5 | 140 | 0.48 | 0.63 |
| >=80 | 3.8 | 8.3 | 3.2 | 0 | 5.0 | 0 | 5.8 | 0 | 4.8 | 0 | 5.8 | 0 | 52.2 | - | 0.21 | 0.37 |
| Hospital volume (%) |  |  |  |  |  |  |  |  |  |  |  |  |  |  |  |  |
| <5 | 33.1 | 66.7 | 29.2 | 36.4 | 37.6 | 18.2 | 50.3 | 77.8 | 44.6 | 76.9 | 33.3 | 40.0 | 0.54 | -40 | 0.96 | 0.14 |
| 5-9 | 18.3 | 8.3 | 19.6 | 18.2 | 23.5 | 18.2 | 40.5 | 0 | 33.3 | 23.1 | 27.7 | 45.0 | 51.4 | 440 | 0.002 | 0.049 |
| 10-14 | 7.6 | 0 | 3.2 | 0 | 13.1 | 27.3 | 0 | 0 | 16.0 | 0 | 23.6 | 5 | 211 | - | <0.0001 | - |
| 15-24 | 15.9 | 8.3 | 23.4 | 18.2 | 25.8 | 36.4 | 9.2 | 22.2 | 6.1 | 0 | 12.4 | 5 | -21.9 | -40.0 | 0.16 | -å |
| >=25 | 25.2 | 16.7 | 24.6 | 27.3 | 0 | 0 | 0 | 0 | 0 | 0 | 3.1 | 5 | -87.8 | -70.0 | <0.0001 | 0.54 |
| Underlying diagnosis (%) |  |  |  |  |  |  |  |  |  |  |  |  |  |  |  |  |
| OA | 60.3 | 58.3 | 57.9 | 36.4 | 64.1 | 9.1 | 70.5 | 66.7 | 66.3 | 53.8 | 60.1 | 60.0 |  |  | 0.94 | 0.93 |
| RA | 10.3 | 0 | 7.6 | 36.4 | 7.4 | 18.2 | 2.9 | 0 | 4.1 | 15.4 | 4.4 | 10 |  |  | 0.0007 | - |
| Fracture | 0 | 0 | 0.6 | 9.1 | 0 | 0 | 0 | 0 | 0 | 0 | 0 | 0 |  |  | - | - |
| Avascular necrosis | 0.7 | 0 | 1.2 | 0 | 0.3 | 9.1 | 0.6 | 0 | 0.7 | 0 | 0.3 | 0 |  |  | 0.60 | - |
| Traumatic Arthropathy | 20.3 | 16.7 | 23.4 | 9.1 | 15.4 | 27.3 | 10.4 | 22.2 | 14.0 | 7.7 | 20.7 | 15 |  |  | 0.90 | 1.0 |
| Other | 8.3 | 25 | 9.4 | 9.1 | 12.7 | 36.4 | 15.6 | 11.1 | 15.0 | 23.1 | 14.4 | 15 |  |  | 0.009 | 0.64 |
| Deyo-Charlson score (%) |  |  |  |  |  |  |  |  |  |  |  |  |  |  |  |  |
| 0 | 74.8 | 83.3 | 72.2 | 45.4 | 74.2 | 72.7 | 74.0 | 77.8 | 70.4 | 69.2 | 70.5 | 60.0 | -5.84 | -28.0 | 0.17 | 0.25 |
| 1 | 23.1 | 8.3 | 26.0 | 36.4 | 21.8 | 18.2 | 22.0 | 22.2 | 19.7 | 15.4 | 23.1 | 35 | -0.04 | 320.2 | 0.99 | 0.20 |
| >=2 | 2.07 | 8.33 | 1.75 | 18.2 | 4.03 | 9.09 | 4.05 | 0 | 9.86 | 15.4 | 6.45 | 5 | 211.6 | -40 | 0.005 | - |

W, White; B, Blacks; OA, osteoarthritis; RA, rheumatoid arthritis
