# Supplementary material for: Racial disparities in total ankle arthroplasty utilization and outcomes
Source: Arthritis Res Ther. 2015 Mar 21;17(1):70. doi: 10.1186/s13075-015-0589-2 (PMC4392624; doi:10.1186/s13075-015-0589-2)
Supplement: Additional file 2: — Is a table presenting TAA outcomes by race from 1998 to 2010. Comparisons of outcomes (mortality, discharge disposition and length of hospital stay) after TAA in Whites and Blacks over the study period from 1998 to 2010 and the change in Whites and Blacks from the first (1998 to 2000) to the last (2009 to 2010) study period. [file 13075_2015_589_MOESM2_ESM.doc]

Additional file 2. TAA outcomes by race from 1998-2010 and change in outcomes over time in Whites and Blacks

|  | 1998-2000 | | 2001-2002 | | 2003-2004 | | 2005-2006 | | 2007 - 2008 | | 2009-2010 | | % Change Last period- first period | |
| --- | --- | --- | --- | --- | --- | --- | --- | --- | --- | --- | --- | --- | --- | --- |
|  | W | B | W | B | W | B | W | B | W | B | W | B | W | B |
| Mortality, n (%) | 0 | 0 | 0 | 0 | 0 | 1 (9.1) | 1 (0.6) | 0 | 0 | 0 | 0 | 0 | - | - |
| Discharge, n (%) |  |  |  |  |  |  |  |  |  |  |  |  |  |  |
| Home | 258 (89.0) | 10 (83.3) | 308 (90.1) | 11 (100) | 262 (87.9) | 7 (63.6) | 149 (86.1) | 8 (88.9) | 242 (82.3) | 10 (76.9) | 503 (85.4) | 17 (85) | -4 | -10 |
| Inpatient facility | 32 (11.0) | 2 (16.67) | 34 (9.94) | 0 | 35 (11.74) | 3 (27.27) | 23 (13.29) | 1 (11.11) | 50 (17.01) | 3 (23.08) | 86 (14.60) | 3 (15) | 32 | 2 |
| Length of stay in days, mean [range] | 2.8 [0, 11] | 3.1 [1, 7] | 2.6 [0, 18] | 2.9 [1, 6] | 2.6 [1, 17] | 9.8 [1, 46] | 2.9 [1, 39] | 3 [1, 6] | 2 [0, 9] | 3 [1, 6] | 2.6 [0, 34] | 2.5 [1, 5] | -9 | -19 |

W, White; B, Blacks
